# Reuterin, Phenyllactic Acid, and Exopolysaccharides as Main Antifungal Molecules Produced by Lactic Acid Bacteria: A Scoping Review

**DOI:** 10.3390/foods13050752

**Published:** 2024-02-29

**Authors:** Andrea Ponzio, Annalisa Rebecchi, Rosanna Zivoli, Lorenzo Morelli

**Affiliations:** 1 Department for Sustainable Food Process, Faculty of Agriculture, Food and Environmental Sciences, Università Cattolica del Sacro Cuore, 29122 Piacenza, Italy; annalisa.rebecchi@unicatt.it (A.R.); lorenzo.morelli@unicatt.it (L.M.); 2 Soremartec Italia S.r.l. (Ferrero Group), P.le P. Ferrero 1, 12051 Alba, Italy

**Keywords:** lactic acid bacteria (LAB), biopreservation, antifungal metabolites, reuterin (Reu), phenyllactic acid (PLA), exopolysaccharides (EPSs)

## Abstract

The primary goal of this scoping review is to collect, analyze, and critically describe information regarding the role of the main compounds (reuterin, phenyllactic acid, and exopolysaccharides) produced by LAB that possess antifungal properties and provide some suggestions for further research. The use of lactic acid bacteria (LAB) to mitigate spoilage and extend the shelf life of foodstuffs has a long history. Recently, there has been a growing interest in the unique properties of these additions to the foodstuffs in which they are applied. In recent studies regarding biopreservation, significant attention has been given to the role of these microorganisms and their metabolites. This fascinating recent discipline aims not only to replace traditional preservation systems, but also to improve the overall quality of the final product. The biologically active by-products produced by lactic acid bacteria are synthesized under certain conditions (time, temperature, aerobiosis, acidity, water activity, etc.), which can be enacted through one of the oldest approaches to food processing: fermentation (commonly used in the dairy and bakery sectors). This study also delves into the biosynthetic pathways through which they are synthesized, with a particular emphasis on what is known about the mechanisms of action against molds in relation to the type of food.

## 1. Introduction

This scoping review deals with a critical analysis regarding a very topical subject of previous research on antifungal activity (highlighting gaps, incompleteness, and errors) and suggestions are made as to the most promising aspects that should be further investigated.

Biopreservation is an interesting topic because there is a growing desire to ensure freshness and safety until the expiry date, reducing food waste; this challenge is linked to economic and sustainability issues. Food loss is not a problem that concerns food products in the strict sense; in fact, it has repercussions along the entire supply chain: from the farm, where the defect (or damage) of the product makes it unusable and, therefore, there has been a waste of water, energy, and other resources, to the last steps before sale, such as proper storage or transport without the waste of packaging, fuel, or energy of various kinds [1].

Related to the concept of food losses and waste is that of food spoilage, i.e., a visual, olfactory, and/or organoleptic change that makes it impossible to consume a food product. The cause can be found in micro-organisms of different types (such as bacteria, molds, and fungi or yeasts), or in larger living organisms such as insects or small animals; in any case, their presence is due to the ideal mix of food source and optimal environmental conditions for their growth [2,3]. In particular, fungi spoilage is responsible for significant food waste and economic losses; worldwide, microbial contamination caused by fungi and toxins is believed to cause problems for about 25% of products in the food sector [4].

Lactic acid bacteria (LAB) are Gram-positive, catalase-negative, non-spore-forming, facultative anaerobic microorganisms used in various food industries (bakery, dairy, fermented meats, vegetables, etc.) [5]. And it is precisely this characteristic versatility that makes them important in the food sector; each of the different LAB species has a greater or lesser potential depending on the application precisely because, depending on the conditions (nutrient sources and environmental conditions), the aforementioned compounds can be synthesized and improve the food in different aspects. Therefore, the interesting variety of metabolites they synthesized during fermentation makes them a promising addition to food; the use of such compounds to reduce the levels of or completely replace antifungal chemical preservatives is the most interesting and current alternative, as these microorganisms are generally recognized as safe (GRAS) [6,7]. It is intriguing how this recent interest goes hand in hand with a very ancient technique: fermentation. In fermentation, microorganisms improve the product’s taste, texture, organoleptic and nutritional values, shelf-life, and safety [8,9].

Biopreservatives with antifungal action are compounds produced by many species, including animals and plants, but this study covers only the ones produced by LAB: organic acids (lactic acid, acetic acid, fatty acids, phenyllactic acid, etc.), reuterin, exopolysaccharides, volatile compounds (for instance, diacetyl), antifungal peptides, hydrogen peroxide (H_2_O_2_), nucleosides, and cyclic dipeptides [10]. In particular, this scoping review focuses on three of the main metabolites: reuterin (Reu), phenyllactic acid (PLA), and exopolysaccharides (EPSs) [11]. The decision to deal with this topic was the result of a thorough literature search (taken from various scientific databases) of the last two decades concerning these metabolites and their possible usefulness in the agro-food world; in order to have a clear overview, some keywords were used for this screening of scientific literature: “BioPreservation”; “Lactic Acid Bacteria”; “Fermentation”; “Food”; “Food Spoilage”; “Antifungal Metabolites”; “Phenyllactic Acid”; “Reuterin”; and “Exopolysaccharides”. The specific choice of these three compounds is the result of an overall assessment of the substances produced as a result of fermentation. They have been chosen because they are the most interesting, promising, and discussed in the last period; because they mainly concern applications in two very relevant food sectors: dairy and baking; and, above all, because the information on them is not yet fully comprehensive. Section 2, Section 3 and Section 4 will analyze the biosynthetic processes, the antifungal effect and mechanism, and the most interesting published studies with their applications.

## 2. Reuterin

Reuterin (Reu) is a broad-spectrum, non-protein, antimicrobial substance whose effect, initially observed only at the antimicrobial level, was observed in the 1980s by Axelsson et al. [12,13]. This initial application of reuterin demonstrated higher effectiveness against Gram-negative microorganisms (*Escherichia coli* and *Salmonella enterica*), which are more sensitive compared to Gram-positive microorganisms (*Listeria monocytogenes* and *Staphylococcus aureus*); only later was its potential as an antifungal agent observed [14,15]. Reu, a bacteriocin named after the manufacturer *Limosilactobacillus reuteri* (the recent new nomenclature registered in the *International Journal of Systematic and Evolutionary Microbiology*), is not the only species that produces reuterin [16]; in fact, the potential to produce this low-molecular-weight compound can be found in some intestinal bacteria such as *E*. *hallii*, *Ruminococcus obeum*, *E. coli*, *Ruminococcus gnavus*, *Flavonifractor prautii*, *Intestinimonas butyriciproducens,* and *Veillonella* spp. [17]. However, it should be noted that these microorganisms are not optimal for reuterin synthesis [18,19].

Many studies have been published on reuterin due to its important biopreservative capabilities. However, some of them contain some misleading information (and this confusion also supports the need for a scoping study such as this one). For example, reuterin has been mistakenly attributed with the Qualified Presumption of Safety status (QPS) in the European Union, but this title should be used to qualify bacterial microorganisms, not metabolites [20].

Today, the term “reuterin” is used to refer to a multi-compound system in which 3-hydroxypropionaldehyde (3-HPA) (primary fraction for what concerns antimicrobial activity), 3-HPA hydrate, 3-HPA dimer, and acrolein are found [17]; the latter component has been indicated as the major contributor to the antimicrobial activity attributed to reuterin [18]. It is known that acrolein has been classified as a class 2A carcinogen by the World Health Organization; therefore, the use of reuterin in food is a cause for concern because of the repercussions on human health [21]. It must always be borne in mind that humans can come into contact with this compound in various situations, as acrolein can be found in what we ingest (food, drink, and even tobacco smoke) or our environment [22].

**Figure 1:**
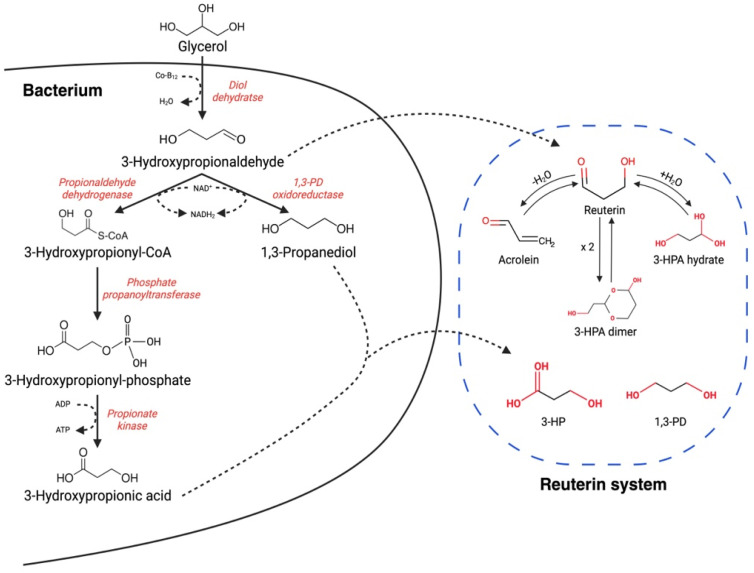
The *pdu* operon metabolic pathway of reuterin and its system.

Glycerol (C_3_H_8_O_3_), a key compound in the process of reuterin synthesis, which is ubiquitous in nature, is essential for the process to begin [23]. Several microorganisms can exploit glycerol as a carbon source [24], which can be utilized through an oxidative or reductive pathway [25]. These series of reactions are made possible by specific genes: those of the 1,2-propanediol utilization operon (*pdu*) (Figure 1). In some *Salmonella* and *Lactobacillus* species, it is possible to trace this particular operon [26,27], and it consists of:

- 1,2-propanediol dehydratase;
- Genes related to vitamin B12 reactivase and regeneration activities;
- CoA-dependent propionaldehyde dehydrogenase (*pduP*);
- Additional enzyme activities [25].

When converting glycerol to reuterin, it is not sufficient to focus on and analyze only the *pdu* operon genes; another cluster responsible for vitamin B12 (*pdu*-*cbi*-*cob*-*hem*) production is essential. In this case, when clusters of genes are considered, the term “gene island” is used to describe the two closely located clusters [28].

**Figure 2:**
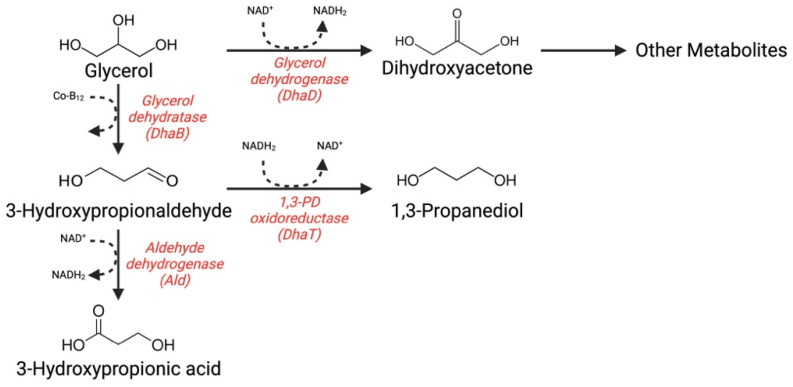
The *dha* operon metabolic pathway of reuterin.

The *pdu* operon genes are not the only genes that make the conversion of glycerol to 3-HP via the 3-HPA intermediate possible; in fact, a second possibility lies in the *dha* operon genes (Figure 2). Unlike the one described above, 3-Hydroxypropionaldehyde (3-HP) is obtained through a reaction that occurs in two steps: first, glycerol is converted to 3-Hydroxypropionaldehyde (3-HPA) by a dehydratase; at a later stage, 3-HP is obtained from a reaction catalyzed by a dehydrogenase. The genes that constitute the *dha* operon fundamentally encode a:

- Glycerol dehydratase (*dhaB*);
- Glycerol dehydratase reactivase (*gdr*);
- 1,3-propanediol oxidoreductase (*dhaT*) [29].

The bacteria that possess such characteristic enzymes are those belonging to the genera of *Enterobacteriaceae*, such as some *Klebsiella*; in addition, it is possible to find them in different gene constructions in other microorganisms, such as some *Lactobacillus* or *Clostridium* [25].

Further work is needed for a better understanding of the mechanism of action of reuterin against bacteria and fungi. Some antimicrobial mechanisms have been analyzed for some components of the reuterin system, such as 3-HPA dimer, 3-HPA (reuterin), or acrolein. In fact, 3-HPA dimer, through competitive inhibition, affects DNA synthesis, blocking the action of ribonucleotide reductase. 3-HPA causes oxidative damage at the protein level by reducing sulfhydryl groups; this is believed to be possible due to the aldehyde groups of reuterin. Acrolein, however, exploiting a Michael addition reaction, also achieves the antimicrobial oxidative result of 3-HPA; in this case, synergistic cooperation with 3-HP has been noted. Although the short-term toxicity tests using in vitro models of the gastrointestinal tract have shown reassuring results [15], further, more complex studies are needed.

In recent decades, several studies have been conducted to obtain naturally preserved food products to meet the increasing consumer demand. Initially, the inhibitory action against bacteria was evaluated. One example is the publication by E. Røssland et al., in which it was noted that, in fermented milk, bacteriocins have no effect on *Bacillus cereus* and, if the pH is not reduced rapidly in the initial phase of the fermentation log, management becomes problematic, as it has the potential to sporulate and exist as endospores [30]. In addition, the article by J.L. Arqués et al. addresses the relationship between reuterin and other LAB-bacteriocins in preventing the growth of foodborne pathogens in milk. The study found that reuterin had a bacteriostatic effect on *Listeria monocytogenes* at the tested temperatures [31].

Furthermore, it has been evaluated and evidence has been provided that the combination production and antifungal activity of reuterin, synthesized by lactic acid bacteria, has a positive effect on silage [32].

Important for applications in food processing is the recently made discovery about reuterin: An LAB strain was found to produce it even under aerobic conditions at low temperatures. In dairy applications, the handling of reuterin has to be carefully controlled so that its strong antifungal power does not affect the cheese-making process when the use of mold is involved in the production [33].

A few studies on the effect of bacteriocins have been conducted, but the behavior of reuterin in food matrices needs further investigation. Recently, the antimicrobial action in fermented milk was evaluated: bacterial and fungal (*Penicillium expanusm*) microbial growth was inactivated [34]. In the wake of fermented dairy products, the effect of reuterin in yogurt was analyzed in 2019, observing a reduction in fungal development (at lower concentrations) and a fungicidal effect (at higher concentrations) [35]. Regarding the actual functioning of the antifungal mechanism of reuterin, theories have been developed: a modification of small molecules and thiol stress groups causes oxidative stress to molds and fungi [36]; a more recent (in silico) study came to the conclusion that the mechanism acts at the enzyme level, inhibiting catalase, catalase-peroxidase, and spore-specific catalase (of *A. flavus*). In particular, reuterin would cause an accumulation of ROS in the cells as a result of competition due to a binding between reuterin and enzyme active sites [37].

Different studies have evaluated the antifungal activity of Reu in vitro at different concentrations, and the results have shown that the minimal inhibitory concentrations (MIC) ranged from 1.89 mM to 7.58 mM and from 4 to 10 mmol/L [38,39].

Reuterin appears to be a promising alternative to chemical additives, especially in the dairy industry, but further studies and evaluations are needed. In particular, it would be beneficial for future research to focus on gaining a deeper understanding of the process by which fungi are inhibited and evaluating the effectiveness of reuterin as an antimicrobial agent when applied to complex food matrices. As already mentioned, one aspect that still raises questions concerns acrolein (or 2-propenal) and the possible occurrence of certain diseases as a result of interaction with the human organism. So far, research that has dealt with the evaluation of cytotoxicity in the reuterine system has not shown any worrying results [15], but the question still exists, and it is urgent to find a reassuring definitive answer because, to date, there are no studies in the existing literature that deal with this danger in the long term.

In conclusion, it would be interesting to evaluate whether, in addition to dairy products, reuterin seems to be the option with the greatest prospects in other food categories; an exciting application to investigate is in plant-based food.

## 3. Phenyllactic Acid

An additional substance whose functions include inhibiting bacterial and fungal development is phenyllactic acid (PLA). Among the metabolites produced by lactic acid bacteria, PLA is one of the cornerstones of antifungal activity. It has been noted that, under high acidity conditions and concentration levels, PLA performs better against fungal growth than lactic acid and acetic acid [40,41].

PLA, or 2-hydroxy-3-phenyl propionic acid (C_9_H_10_O_3_), is a natural organic acid resulting from the metabolism of phenylalanine (*Phe*) [42]. At the microbial level, the synthesis of this important broad-spectrum antimicrobial compound was first examined in 1998 in *Geotrichum candidum* against the growth of *L. monocytogens* [43]. PLA biosynthesis has also been observed, at a later stage, in microorganisms other than LAB, such as exploited *E. coli*; in fact, such biosynthesis is made possible by a specific gene (D-lactate dehydrogenase), the lack of which makes PLA production impossible [44,45].

Luo et al. reported that the D-PLA isoform is more intriguing than the L-PLA isoform due to its potential as an antimicrobial and antiseptic agent, thermal stability, effectiveness, dynamic behavior within a wide pH range, and solubility in water. These properties apply to the food, feed, and pharmaceutical fields [46]. Over the years, several studies have been conducted (especially in leavened bakery products) on this compound, whose presence in fermented food products is not uncommon [10]. In fact, research has shown that an inhibiting action against *Penicillium expansum*, *P*. *verrucosum*, *P*. *citrinum*, *P. roqueforti*, *Aspergillus flavus*, *A. ochraceus A. niger*, *Monilia sitophila*, *Fusarium graminearum*, *Candida pulcherrima*, etc., occurs [47].

However, the antifungal efficacy of PLA is still a matter of debate: the amount present in some natural foods would appear to be insufficient for bio-preservation purposes [48]. Even with regard to minimal inhibitory concentration (MIC) values, further studies will be necessary, as more, or less, interesting values (3–300 mM) can be obtained depending on the matrix and the level of environmental acidity [49]. For example, it has been noted that PLA is characterized by an MIC value that varies depending on the microorganism it is tested against: for *Aspergillus fumigatus,* it is 2.5 mg/mL; in contrast, against *Aspergillus niger,* the value is 20 mg/mL [50,51].

**Figure 3:**
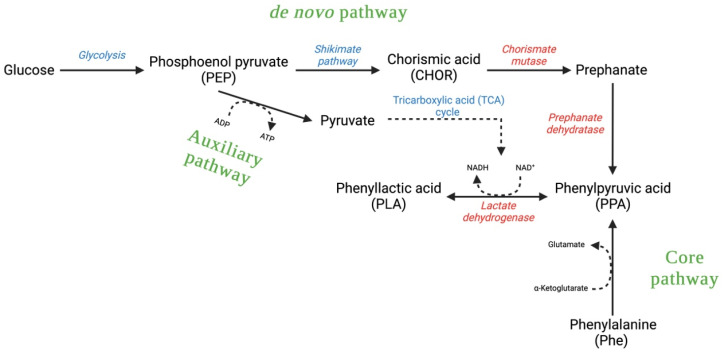
The three biosynthetic pathways of phenyllactic acid.

Main LAB species producing PLA include *Lactiplantibacillus plantarum*, *Companilactobacillus crustorum*, *Lentilactobacillus buchneri*, *Limosilactobacillus reuteri*, *Levilactobacillus brevis*, *Lacticaseibacillus casei*, *Lactobacillus delbrueckii*, *Pediococcus acidilactici*, and *Weissella confusa* [52,53]. To analyze the process of PLA synthesis by LAB, three pathways have been identified through a multi-omics analysis (Figure 3):

- Core pathway—the one that has been most studied;
- De novo biosynthetic pathway or central carbon metabolism;
- Auxiliary pathway [49].

Again, there is not much information regarding how the antifungal mechanism works. At present, it is believed that, because of an interaction with the proton gradient, phenyllactic acid (with the help of other metabolites) may have an inhibitory effect at the level of cellular enzymes [11].

PLA is still a metabolite under discussion regarding its potential as a bioconservative; some of the existing literature ranks it among the most interesting organic acids in spoilage inhibition [54,55], and others claim that its contribution is irrelevant to mold development [56]. Several studies have investigated this metabolite, and compared to other compounds, more data can be found on food as a complex matrix. In a recent study, through the addition of PLA, an inhibiting effect against *Penicillium paneum* and *Aspergillus niger* was observed in bread production [41]. The limiting aspects of this substance for preservative purposes are bacterial production inefficiency and a high cost at the producer level [57]. Although it is a much-treasured metabolite, theories and models have been elaborated as to the mechanism by which PLA inhibits fungal growth: suppression of the sporulation phase and radial growth [49].

Therefore, future studies should investigate the production (identifying optimal conditions), mechanism of action, and stability of this substance against molds while continuing to work on processes and/or finished products to obtain more representative results. In contrast to other promising substances in the field of biopreservation, a safety assessment of PLA has been carried out, finding this metabolite to be non-toxic (and not to cause olfactory level changes) for animals and humans [58].

Given the growing interest, this should be applied not only to traditional doughs and bakery products, but also to sourdoughs generated from different raw materials; the highlight of this compound is its stability during baking [59].

Therefore, the future objective is to verify the actual antifungal potential and the sensory and organoleptic evaluation of the final product. The importance of determining the minimum inhibitory concentration of PLA for the main type of fungi in relation to the specific product it is used on should not be overlooked.

## 4. Exopolysaccharides

Related to biopreservation, there has been a recent increase in interest in another class of compounds of microbial origin: exopolysaccharides (EPSs), which are secondary metabolites produced mainly during bacterial growth [60]. The name “exopolysaccharide” was designated in 1967 to refer to high-molecular-weight (HMW) carbohydrates secreted into the environment by microorganisms. EPSs are considered extracellular polymers, as 40–95% of extracellular polymers include polysaccharides [61].

This scoping study focuses on microbial-derived EPSs, particularly those produced by lactic acid bacteria. LAB can synthesize and release these degradable biopolymers, whose basic units are sugar monosaccharides, into the extracellular environment under certain conditions [62]. Exopolysaccharide biosynthesis is influenced by the presence or absence of carbon and nitrogen sources, acidity, temperature, oxygen availability, and cellular concentration [63].

Exploiting EPS’s technological and functional properties is a promising tool in industrial food application. These long-chain polysaccharides lend themselves as structural modifiers due to their water-holding capacity; moreover, given their beneficial effect, they are also a prebiotic, anti-inflammatory, anti-cancer, antioxidant, and antiviral alternative [64]. An additional and interesting application is to exploit these compounds to form films for biodegradable packaging [65].

Recently, there has been increased interest in the use of metal nanoparticles in response to drug resistance by pathogens. Recent research has assessed this possibility, given the potential of these innovative alternatives due to their environmentally friendly synthesis and broad spectrum of action against various microorganisms. This topic is related to exopolysaccharides as an excellent substrate for the synthesis of the nanoparticles tested, and they have been shown to have good antimicrobial activity against Gram-positive and Gram-negative bacteria as well as fungi [66].

**Figure 4:**
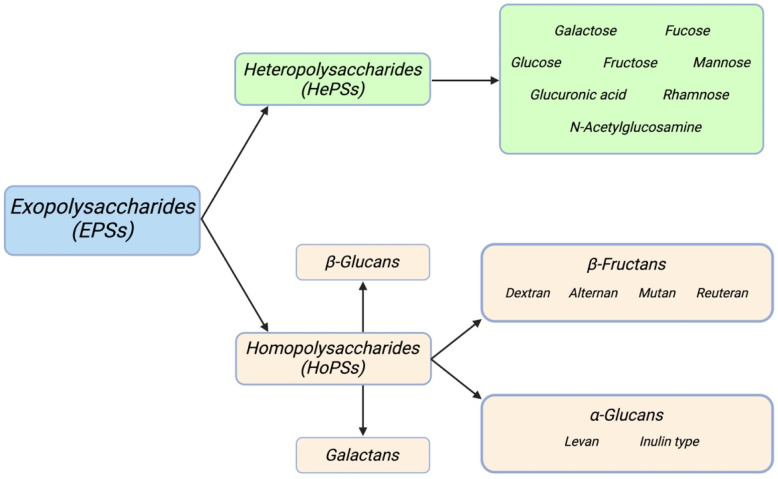
Classification of exopolysaccharides of bacterial origin.

However, the main reason they are discussed in this review is their potential application for antifungal purposes, but it would be of significant interest to study and correlate the presence of these metabolites with advantages and benefits on final products. Taking chemical structure as a reference, exopolysaccharides can be divided into two classes (Figure 4): homopolysaccharides (HoPSs) and heteropolysaccharides (HePSs) [67]. This classification is based on the composition of the sugar unit: in HoPSs, the residual sugar present is of a single type; in contrast, HePSs are polymers formed from different types of monosaccharides [68,69].

As for HoPSs, there are four groups into which they can be subdivided according to the position and type of bonding of the carbon involved:

- α-glucans, in which dextran (α-1,6), alternans (1,3 α and α-1,6), mutans (1,3 α and α-1,6), and reuterans (1,4 and α-1,6) can be distinguished;
- β-glucans, only one type linked via β-1,2, β-1,3, and β-1,4 glycosidic bonds;
- β-fructans, formed by the subgroups of inulin-type (β-2,1) and levan (β-2,6);
- Galactans: bonds that characterize this rarer class produced by *Lactobacillus* spp. are β-1,3 or β-1,6 [70,71].

Microorganisms capable of producing this type of polymer include *Limosilactobacillus fermentum*, *Lactobacillus johnsonii*, and *Leuconostoc mesenteroides* [72].

Turning to HePSs, most LAB-EPSs (lactic acid bacteria-derived exopolysaccharides) fall into this group. Usually, these are chains composed of three to eight units of sugars, such as glucose, galactose, or rhamnose. The three sugars just mentioned are not the only ones present; other monosaccharides such as fructose, mannose, fucose, glucuronic acid, and N-acetyl-glucosides can be found [73].

It has been noted that there are several bacteria capable of producing such exopolysaccharides; among them are *Lactococcus lactis*, *Lactobacillus rhamnosus*, *Lacticasei-bacillus casei*, *Lactobacillus delbrueckii*, *Lactobacillus bulgaricus*, *Lactobacillus acidophilus*, *Lactobacillus helveticus*, and *Streptococcus* [74].

**Figure 5:**
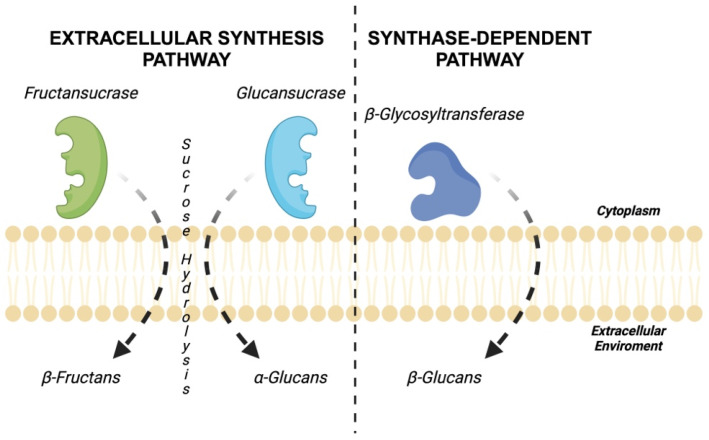
The extracellular and the synthase-dependent pathway of HoPSs.

Moreover, another issue involving exopolysaccharides is complex, partly because of the large number of gene products involved in biosynthesis [75]. To date, four pathways for the synthesis of LAB-EPSs have been identified: the extracellular synthesis pathway, the synthase-dependent pathway, the ATP-Binding Cassette (ABC) transporter-dependent pathway, and the Wzx/Wzy-dependent pathway. Of those mentioned, the two most exploited pathways are the extracellular synthesis (Figure 5) and the Wzx/Wzy-dependent synthesis pathways [71,76].

**Figure 6:**
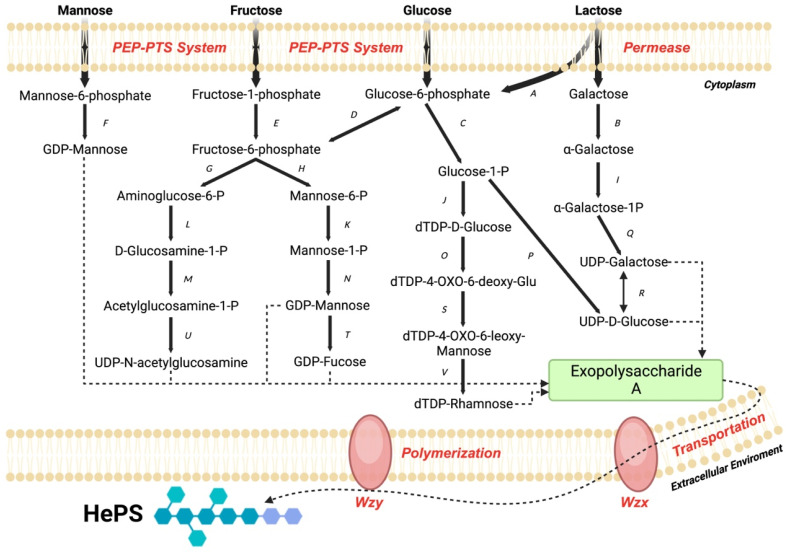
The Wzx/Wzy-dependent pathway of HePSs. A: β-Galactosidase and Glucokinase; B: Galactose mutarotase; C: α-Phosphoglucomutase; D: Glucose-6-phosphate isomerase; E: 1-phosphofructokinase; F: Phosphomannomutase and Mannose 1-phosphateguanylystransferase; G: Glucosamine-6-phosphate deaminase; H: Mannose-6-phosphate isomerase; I: α-Galactokinase; J: Glucose-1-phosphate thymidylyltransferase; K: Phosphomannomutase; L: Phosphoglucosamine mutase; M: UDP-N-acetylglucosamine pyrophosphorylase; N: Mannose 1-phosphateguanylystransferase; O: dTDP-glucose 4,6-dehydratase; P: UTP-glucose-1-phosphate uridylyltransferase; Q: UDP-glucose–hexose-1-phosphate uridylyltransferase; R: UDP-glucose 4-epimerase; S: dTDP-4-dehydrorhamnose 3,5-epimerase; T: GDP-mannose-4,6-dehydratase; U: UDP-glucose 4-epimerase; V: dTDP-4-dehydrorhamnose reductase.

The Wzx/Wzy-dependent pathway of HePSs is a complex process consisting of five steps (Figure 6). It is a complicated mechanism that differs according to the starting sugar: mannose, fructose, glucose, or lactose [76,77].

In addition to the synthase-dependent pathway and the Wzx/Wzy-dependent pathway, there is a third intracellular pathway: the ATP-Binding Cassette (ABC) transporter-dependent pathway. This relates more to capsular polysaccharides (CPSs), and being anchored to the cell surface, they are not considered true exopolysaccharides [78].

In the work of Wu et al., three possible points where EPSs show inhibitory effects against fungi were identified: destruction of cell and plasma membrane, altering cell division, or degrading DNA, whereas in 2018, in the publication by Abinaya et al., it was noted that, depending on the permeability of the cell membrane, EPSs can affect the respiratory chain, causing fungal cell death [79].

Linked to membrane permeability, polysaccharides’ possibility of barrier action to inhibit the development of fungi and bacteria was investigated. As the concentration of EPS increases, the incoming amount of nutrients and substances essential for pathogen survival should decrease [80].

An additional target of EPS has been identified against biofilms. Polysaccharides can reduce the possibility of fungal adhesion, hydrophobicity (destabilizing the structure), mobility, and cellular interactions for biofilm production [60,79]. Recently, the activity against biofilms by EPSs was investigated in depth by Wang et al., and it was found that these metabolites synthesized by LAB are capable of modifying bacteria and fungi at the cellular level, suppressing the anchoring activity of pathogens, and also affecting this process at the gene level [81,82].

EPS synthesized from certain *Lactobacillus* strains show intriguing potential in the fungal inhibition of certain *Candida* species [83,84]; the presence of lactic acid bacteria and the substances they produce is able to provide protection to oral epithelial cells by preventing fungal adhesion [85].

The minimum inhibitory concentration (MIC) of EPS was evaluated in a recent study, in which (after purification) different concentrations were tested against different microorganisms (*P. aeruginosa*, *E. coli*, *S. aureus*, *B. subtilis*, *C. albicans*, and *A. niger*). This resulted in an MIC value between 15 µg/mL and 52 µg/mL, depending on the bacterium or mold [86].

Exopolysaccharides are best known for the properties they can impart to the products in which they are applied, especially at the structural level. The most promising sectors for their application for biopreservative purposes are bakery and dairy, but knowledge of the mechanism in its entirety is still limited. Likewise, work on the application in bakery products would require more in-depth investigation, especially due to the thermostability (given the treatment to which such foods are usually subjected). One of the most recent and interesting works on biopreservation in a bakery was published by Illueca, Francisco, et al. in 2023; in the article, the antifungal power of LAB applied to sourdough is evaluated, and the topic is explored from different aspects [87].

An article such as the one just mentioned should be taken as an example, as there are not many that channel and relate, within the same work, the different facets, such as LAB-substrate interaction, the fermentation process with its metabolomic analysis, the thermostability of the synthesised compounds and the inhibitory power of the most representative fungi.

An interesting peculiarity of future work could be to perform a complete screening of the exopolysaccharides in a given food matrix and subsequently understand whether there are compounds that have more or less relevance at the antifungal level.

## 5. Conclusions

The challenge in the agri-food chain is always the same: to meet consumer demand. Today, the consumer pays more attention to the product when purchasing, preferring clean labels; zero-kilometer, organic products; or brands committed to combating food waste. Following this trend, the term “sustainability” is currently being abused and applied to different areas. Although it is a hot topic as a global need, in the food sector, economic assessments must also be made in parallel to see whether it makes sense to bear the costs for that type of production.

One of the most appealing possibilities for improvement in the food sector is biopreservation, i.e., the development and implementation of natural processes such as fermentation and the related use of microorganisms, in order to achieve a reduction in the use of chemical preservatives without affecting shelf-life too much, but also improvements in terms of technology, organoleptic properties, and safety. The microorganisms most commonly used for these applications include lactic acid bacteria, and the changes that can be achieved in the product are due to the variety of substances synthesized during the fermentation process: organic acids, acids derived from amino acids, reuterin system, cyclic dipeptides, fatty acids, and volatile compounds. Depending on the starting matrix, not only are each of these substances produced in different quantities, but the effect on the food can also differ. This adaptability makes LAB even more advantageous in the agri-food sector. The microorganisms most commonly used for these applications belong to the lactic acid bacteria category, and the changes in the product are due to the very compounds they can synthesize and release during fermentation.

**Figure 7:**
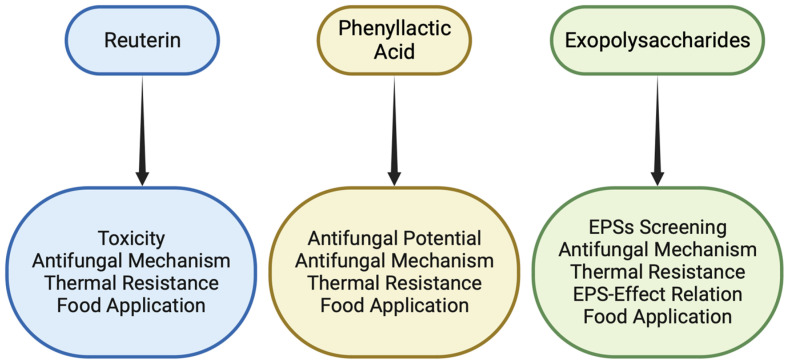
Overview of properties requiring further investigation.

The aspect that arouses so much interest in this area of research is the promising inhibitory effect these substances can have on bacteria and fungi. Important progress has been made in recent years; however, further studies are needed to fully understand how these metabolites work (Figure 7). In particular, the future challenge concerning these compounds is to discover every single step of the mechanism by which fungal development is inhibited. Once this automatism is understood, it will finally be possible to improve research efficiency by applying the best compound to the relative food for which it performs best.

To date, two of the aspects that need to be better known concern the verification of the possible repercussions (positive and negative) on the human organism following prolonged ingestion of these substances; but as already mentioned, it is no less important to know the entire process of action of molecules with antifungal power in order to be able to exploit it to its full potential.

Once a good understanding of the behavior of these substances is obtained, applications in the food system can be diverse, e.g., by incorporating them into packaging technologies. The importance of fermentation for biopreservation is fundamental; this technique, once understood and exploited to its full potential, will provide the solution to two of the main challenges facing the agri-food sector today: the reduction in chemical preservatives by not excessively altering shelf life and the reduction in food waste due to fungal spoilage.

In conclusion, reviews that delve into and critically examine (highlighting gaps and inaccuracies) an emerging topic such as this are increasingly useful in bringing together the fragmented information in the literature and are an asset in understanding where to direct and optimize the focus of future research.

## Data Availability

The original contributions presented in the study are included in the article, further inquiries can be directed to the corresponding author.
